# A platform for detecting cross-resistance in antibacterial drug discovery

**DOI:** 10.1093/jac/dkab063

**Published:** 2021-03-23

**Authors:** Luiza H Galarion, Merianne Mohamad, Zeyad Alzeyadi, Christopher P Randall, Alex J O’Neill

**Affiliations:** School of Molecular and Cellular Biology, Faculty of Biological Sciences, University of Leeds, Leeds LS2 9JT, UK; School of Molecular and Cellular Biology, Faculty of Biological Sciences, University of Leeds, Leeds LS2 9JT, UK; School of Molecular and Cellular Biology, Faculty of Biological Sciences, University of Leeds, Leeds LS2 9JT, UK; School of Molecular and Cellular Biology, Faculty of Biological Sciences, University of Leeds, Leeds LS2 9JT, UK; School of Molecular and Cellular Biology, Faculty of Biological Sciences, University of Leeds, Leeds LS2 9JT, UK

## Abstract

**Background:**

To address the growing antibiotic resistance problem, new antibacterial drugs must exert activity against pathogens resistant to agents already in use. With a view to providing a rapid means for deselecting antibacterial drug candidates that fail to meet this requirement, we report here the generation and application of a platform for detecting cross-resistance between established and novel antibacterial agents.

**Methods:**

This first iteration of the cross-resistance platform (CRP) consists of 28 strains of defined resistance genotype, established in a uniform genetic background (the SH1000 strain of the clinically significant pathogen *Staphylococcus aureus*). Most CRP members were engineered through introduction of constitutively expressed resistance determinants on a low copy-number plasmid, with a smaller number selected as spontaneous resistant mutants.

**Results:**

Members of the CRP collectively exhibit resistance to many of the major classes of antibacterial agent in use. We employed the CRP to test two antibiotics that have been proposed in the literature as potential drug candidates: γ-actinorhodin and batumin. No cross-resistance was detected for γ-actinorhodin, whilst a CRP member resistant to triclosan exhibited a 32-fold reduction in susceptibility to batumin. Thus, a resistance phenotype that already exists in clinical strains mediates profound resistance to batumin, implying that this compound is not a promising antibacterial drug candidate.

**Conclusions:**

By detecting cross-resistance between established and novel antibacterial agents, the CRP offers the ability to deselect compounds whose activity is substantially impaired by existing resistance mechanisms. The CRP therefore represents a useful addition to the antibacterial drug discovery toolbox.

## Introduction

It is widely accepted that new antibacterial drugs will be required to address the growing problem of antibiotic resistance in pathogenic bacteria.^1^ ‘New’ in this context is often understood to mean unprecedented in terms of chemical structure and/or antibacterial mode of action and it is certainly the case that useful antibacterial drug candidates will likely possess one or both of these attributes. Nevertheless, these attributes are proxies for the type of novelty that is ultimately required: the ability to exert an antibacterial effect on pathogens resistant to drug classes already in clinical use. After all, an antibacterial drug candidate that possesses chemical and/or mechanistic novelty will have limited utility if its activity is substantially comprised as a result of cross-resistance with earlier classes.

Despite this, little direct effort is expended on investigating/avoiding cross-resistance in the typical antibacterial discovery project. Instead, the potential for cross-resistance to newly discovered scaffolds is usually addressed indirectly—and often at a relatively advanced stage of preclinical evaluation—by assessing the activity of the compound against sizeable collections of target pathogen(s) isolated from the clinic.^2^ An exception to this approach is in discovery efforts that seek to identify novel analogues of an established antibacterial drug class, since such projects must proceed alive to the potential for cross-resistance from the very outset. In these cases, analogues are tested at an early stage of evaluation against strains harbouring resistance determinants known to compromise the antibacterial activity of clinically deployed class members (e.g. Seiple *et al*.^3^).

We consider that this latter strategy could usefully be employed more broadly in antibacterial discovery to provide a rapid and direct indication of potential cross-resistance issues at an early stage in the process, thereby reducing wasted effort in progressing compounds that are only later revealed to possess such resistance liabilities. Here, we describe the initial iteration of a platform for cross-resistance testing, comprising a panel of *Staphylococcus aureus* strains of defined antimicrobial resistance genotype established in a uniform genetic background. Use of this cross-resistance platform (CRP) to test two potential antibacterial drug candidates revealed that the activity of one of these (batumin) is dramatically attenuated by a resistance phenotype that pre-exists in the clinic, implying that it is not a promising candidate for antibacterial chemotherapy, and underscoring the utility of the proposed approach to cross-resistance testing.

## Materials and methods

### Generation of the CRP

Horizontally acquired antibiotic resistance genes were in most cases amplified by PCR and cloned in *Escherichia coli* using a modified version of shuttle vector pSK5487^4^ termed pSK5487M (Figure S1, available as Supplementary data at *JAC* Online), downstream of the constitutive *qacR* promoter.^5^ Where appropriate template DNA for PCR amplification was not available, DNA corresponding to resistance genes was instead obtained by synthesis (Genewiz). All PCR amplicons and synthesized DNA encompassed the native ribosome-binding sites of resistance determinants and most introduced BstBI-restriction sites at the termini of the fragments for ligation into BstBI-digested pSK5487M (the exception being resistance determinants whose sequence included an internal BstBI site, which were instead ligated into pSK5487M by blunt-end cloning at the blunted BstBI site). Constructs established in *E. coli* were subjected to DNA sequencing before electroporation into the restriction-deficient staphylococcal cloning host RN4220,^6^ with subsequent recovery and electroporation into *S. aureus* SH1000.^7^ Introduction of pSK5487M: *mecA* into SH1000 did not result in a detectable change in oxacillin MIC, a phenomenon attributable to the fact that only a minority subpopulation of artificially generated *mecA*+ strains usually expresses resistance;^8^ homogeneous/overt resistance was subsequently selected in this strain by plating onto agar containing oxacillin at 100 mg/L as described previously.^8^^,^^9^

Several strains exhibiting resistance to antibacterial agents as a result of mutation were isolated and characterized in previous studies (AJUL22,^10^ AJUL26/AJUL27^11^). Strains with mutational resistance to rifampicin and triclosan were selected on agar/by serial passage, respectively, and characterized in the former case by PCR amplification and DNA sequencing of *rpoB* and in the latter case by WGS according to established methodology;^12^ the mutants ultimately chosen for inclusion in the CRP carry resistance mutations commonly found in clinical isolates^13^^,^^14^ (whilst the FabI_D101G_ substitution in our triclosan-resistant mutant does not appear to have been detected in clinical isolates, the -C_34_T and -T_109_G mutations upstream of the *fabI* gene have both independently been reported to mediate resistance in such strains through increased FabI expression). Strain AJUL25, which exhibits resistance to sulfamethoxazole as a result of two common resistance mutations in the *dhps* gene,^15^ was created by *ϕ*80-mediated transduction of this locus from a strain (*S. aureus* Newman) that naturally harbours these.

### Susceptibility testing

MIC determinations were generally performed by broth microdilution in CAMHB, according to CLSI guidelines. Exceptions were made in isolated cases to improve discrimination between susceptible and resistant strains. For sulfamethoxazole, the bacterial inoculum was reduced 10-fold (to 5 × 10^4^ cfu), whilst susceptibility testing with fusidic acid was conducted by agar dilution using CAMHA. Antibacterial agents were from Sigma–Aldrich, with the exception of linezolid and quinupristin/dalfopristin (both from Cambridge Bioscience) and mupirocin (PanReac AppliChem).

## Results and discussion

The basic design principles of the CRP are as follows. This initial iteration of the platform was established in a Gram-positive bacterium to offer the broadest utility, since the vast majority of antibacterial drug candidates exhibit anti-Gram-positive activity (by contrast, only a small minority are active against Gram-negative bacteria). Accordingly, we generated the CRP in the important Gram-positive pathogen *S. aureus*, employing the well-characterized and -behaved laboratory strain, SH1000.^7^

Each member of the CRP possesses a defined resistance genotype. Only one strain in the collection has been intentionally engineered to carry more than one type of resistance determinant (AJUL17; to provide simultaneous resistance to group A and B streptogramins and hence to the combination drug quinupristin/dalfopristin), though all strains carrying cloned resistance genes also harbour the selectable marker (*cat*) intrinsically present on pSK5487M and are therefore additionally resistant to chloramphenicol. For the most part, expression of cloned resistance determinants in the CRP is driven from a low/moderate strength, constitutive promoter (P_*qacR*_). This approach sought to address the fact that a number of staphylococcal resistance determinants (e.g. *bla, erm, mec*) ordinarily require induction for the resistance phenotype to manifest and failure to induce resistance in the test would prevent detection of cross-resistance. However, for a small number of resistance genes (strains AJUL5, AJUL10, AJUL14 and AJUL20), the level of resistance observed following expression from P_*qacR*_ was only modest or negligible, and, in such cases, the determinant was re-cloned with its native expression signals.

The resistance genotypes and phenotypes of the CRP are given in Table 1. In some cases (e.g. for determinants known to mediate resistance to more than one antibacterial drug class), susceptibility data for several antibacterial agents are shown. All CRP members exhibited at minimum a 4-fold decrease in susceptibility to at least one corresponding antibacterial agent.

**Table 1.** Nature of the strains constituting the CRP described in this study

Antibacterial class to which resistance is mediated		Strain and resistance genotype		Reference accession number for resistance determinant	Resistance phenotype		
					agents tested	MIC (mg/L)	SH1000 MIC (mg/L)
Protein synthesis inhibitors	phenicols	AJUL1	SH1000 (pSK5487M) [empty vector]		chloramphenicol	64	4
	aminoglycosides	AJUL2	SH1000 (pSK5487M: *aacA-aphD*) [bifunctional *aac(6')-aph(2")*]	WP_001028144.1	gentamicin	8	0.5
					kanamycin	32	0.5
					neomycin	0.5	0.5
					tobramycin	8	0.5
		AJUL3	SH1000 (pSK5487M: *aadD*)	WP_137075613.1	gentamicin	0.5	0.5
					kanamycin	>512	0.5
					neomycin	8	0.5
					tobramycin	256	0.5
		AJUL4	SH1000 (pSK5487M: *ant(9)-Ia*)	WP_000067268.1	spectinomycin	>512	64
		AJUL5	SH1000 (pSK5487M: *aph(3’)-IIIa*)	EGQ1519538.1	kanamycin	64	0.5
					neomycin	32	1
		AJUL6	SH1000 (pSK5487M: *str*)	AYK28244.1	streptomycin	16	1
	phenicols, lincosamides, oxazolidinones, pleuromutilins, streptogramins (A)	AJUL7	SH1000 (pSK5487M: *cfr*)	ARQ19305.1	florfenicol	128	8
					linezolid	8	2
					lincomycin	>512	0.5
					retapamulin	16	0.0625
	macrolides, lincosamides, streptogramins (A)	AJUL8	SH1000 (pSK5487M: *ermB*)	QCY67633.1	erythromycin	>512	0.5
		AJUL9	SH1000 (pSK5487M: *ermC*)	AIU96746.1	erythromycin	>512	0.5
		AJUL10	SH1000 (pSK5487M: *msrA*)	WP_002447408	erythromycin	16	0.5
	fusidic acid	AJUL11	SH1000 (pSK5487M: *fusB*)	WP_000855537.1	fusidic acid	4	0.016
	mupirocin	AJUL12	SH1000 (pSK5487M: *mupA*)	WP_000163435.1	mupirocin	16	0.125
	oxazolidinones and phenicols	AJUL13	SH1000 (pSK5487M: *optrA*)	AON96416	linezolid	4	2
					tedizolid	4	0.5
					florfenicol	32	4
	tetracyclines	AJUL14	SH1000 (pSK5487M: *tetK*)	WP_031903778	tetracycline	64	0.5
		AJUL15	SH1000 (pSK5487M: *tetM*)	QGQ78162.1	tetracycline	32	1
	pleuromutilins	AJUL16	SH1000 (pSK5487M: *vga(A)*_LC_)	AQY75653.1	retapamulin	1	0.0625
	streptogramins (A, B)	AJUL17	SH1000 (pSK5487M: v*ga(A), ermC*)	*vga(A)*: WP_032489639; *ermC*: AIU96746.1	quinupristin/dalfopristin	1	0.125
Peptidoglycan synthesis inhibitors	bacitracin	AJUL18	SH1000 (pSK5487M: *bcrABD*)	CP030662.1	bacitracin	>512	64
	β-lactams (penicillinase-susceptible)	AJUL19	SH1000 (pSK5487M: *blaZ*)	QGQ78449.1	penicillin G	32	0.031
	β-lactams (penicillinase-stable)	AJUL20	SH1000 (pSK5487M: *mecA*)	QIE05029.1	oxacillin	512	0.125
	fosfomycin	AJUL21	SH1000 (pSK5487M: *fosB*)	WP_011276918	fosfomycin	>512	16
Membrane active agents	daptomycin	AJUL22	SH1000 [MprF_S295L_]	–	daptomycin	8	2
RNA polymerase inhibitors	rifamycins	AJUL23	SH1000 [RpoB_H481Y_]	–	rifampicin	>512	2
Folate synthesis inhibitors	diaminopyrimidines	AJUL24	SH1000 (pSK5487: *dfrK*)	WP_012779617.1	trimethoprim	512	2
	sulphonamides	AJUL25	SH1000 [DHPS_F17L, E208K_]	–	sulfamethoxazole	512	64
DNA replication inhibitors	fluoroquinolones	AJUL26	SH1000 [GrlA_S80Y_, GyrA_S84L_]	–	norfloxacin	32	4
	aminocoumarins	AJUL27	SH1000 [GyrB_G85S_, _D89G_]	–	novobiocin	32	1
Fatty acid biosynthesis inhibitors	triclosan	AJUL28	SH1000 [-T_109_G and -C_34_T in *fabI* promoter region, FabI_D101G_]	–	triclosan	2	0.0625

To illustrate the potential utility of the CRP for the evaluation of antibacterial drug candidates, we describe its use to test two antistaphylococcal agents that may have therapeutic potential: γ-actinorhodin^16^ and batumin (kalimantacin A).^17^ For the former compound, all members of the CRP showed the same level of susceptibility as the parent strain (2 mg/L γ-actinorhodin), indicating an absence of cross-resistance in this panel of strains. Whilst this result does not exclude the possibility that cross-resistance to γ-actinorhodin exists and/or could arise in clinical isolates, it does provide some reassurance that the antibacterial activity of this compound will not be abrogated by a common resistance determinant. For batumin, a single strain in the CRP exhibited a reduction in susceptibility to the compound relative to SH1000; the triclosan-resistant strain AJUL28 showed a 32-fold decrease in susceptibility (8 versus 0.25 mg/L). Thus, a resistance phenotype that already exists in the clinic^13^ provides cross-resistance to batumin. We corroborated this observation by demonstrating that several triclosan-resistant clinical isolates and laboratory-generated mutants all exhibited substantial reductions in susceptibility to batumin (data not shown). Whether evidence of pre-existing cross-resistance should preclude further development of an antibacterial drug candidate will warrant careful consideration on a case-by-case basis, considering—amongst other aspects—the level and clinical prevalence of the resistance in question. In the case of batumin, existing triclosan resistance mediates a profound reduction in susceptibility to the compound and is not uncommon amongst clinical isolates;^18^ on this basis, batumin is probably not a promising antistaphylococcal drug candidate.

Beyond its use to rule antibacterial drug candidates from further consideration, detection of cross-resistance using the CRP can also provide additional insight into antibacterial agents undergoing evaluation. Until recently, the mode of action of batumin remained poorly characterized, though limited evidence suggested that it involves inhibition of fatty acid biosynthesis (FAB).^19^^,^^20^ The finding that promoter mutations causing increased expression of the FAB gene *fabI*^13^ confer reduced susceptibility to batumin further reinforces the idea that this compound acts on FAB and indeed implicates FabI as a plausible target. Whilst the present work was being readied for publication, Fage *et al*.^21^ confirmed that FabI is indeed the target of batumin.

### Conclusions

Engineered antibiotic-resistant bacteria are already in use in drug discovery projects to achieve dereplication of natural products (i.e. deselection of known chemical scaffolds).^22^^,^^23^ The purpose of the CRP is somewhat distinct, aimed instead at deselecting compounds whose activity is impaired by known resistance mechanisms; in other words, the CRP seeks to effect dereplication at the biological level, rather than the chemical level. Accordingly, the types of resistance determinant used in these two approaches differ, with the former focussing on those that reduce susceptibility to common natural-product antibiotics and the CRP employing resistance genes or mutations commonly found in clinical isolates. Nevertheless, the two approaches are complementary and one could envisage a future platform comprising a far more extensive/near-comprehensive set of known antibiotic resistance determinants to achieve both ends simultaneously.

We consider that the CRP represents a useful addition to the antibacterial drug discovery toolbox and have therefore made it available to researchers through BEI Resources (https://www.beiresources.org; Resource NR-55306). We welcome additions to the platform that follow the same basic design principles, ideally employing the same cloning vehicle/host to ensure uniformity.

## Supplementary Material

dkab063_Supplementary_Data
