# Supplementary figures and images for: A platform for detecting cross-resistance in antibacterial drug discovery
Source: J Antimicrob Chemother. 2021 Mar 23;76(6):1467–71. doi: 10.1093/jac/dkab063 (PMC8843079; doi:10.1093/jac/dkab063)

**Supplementary data**

**
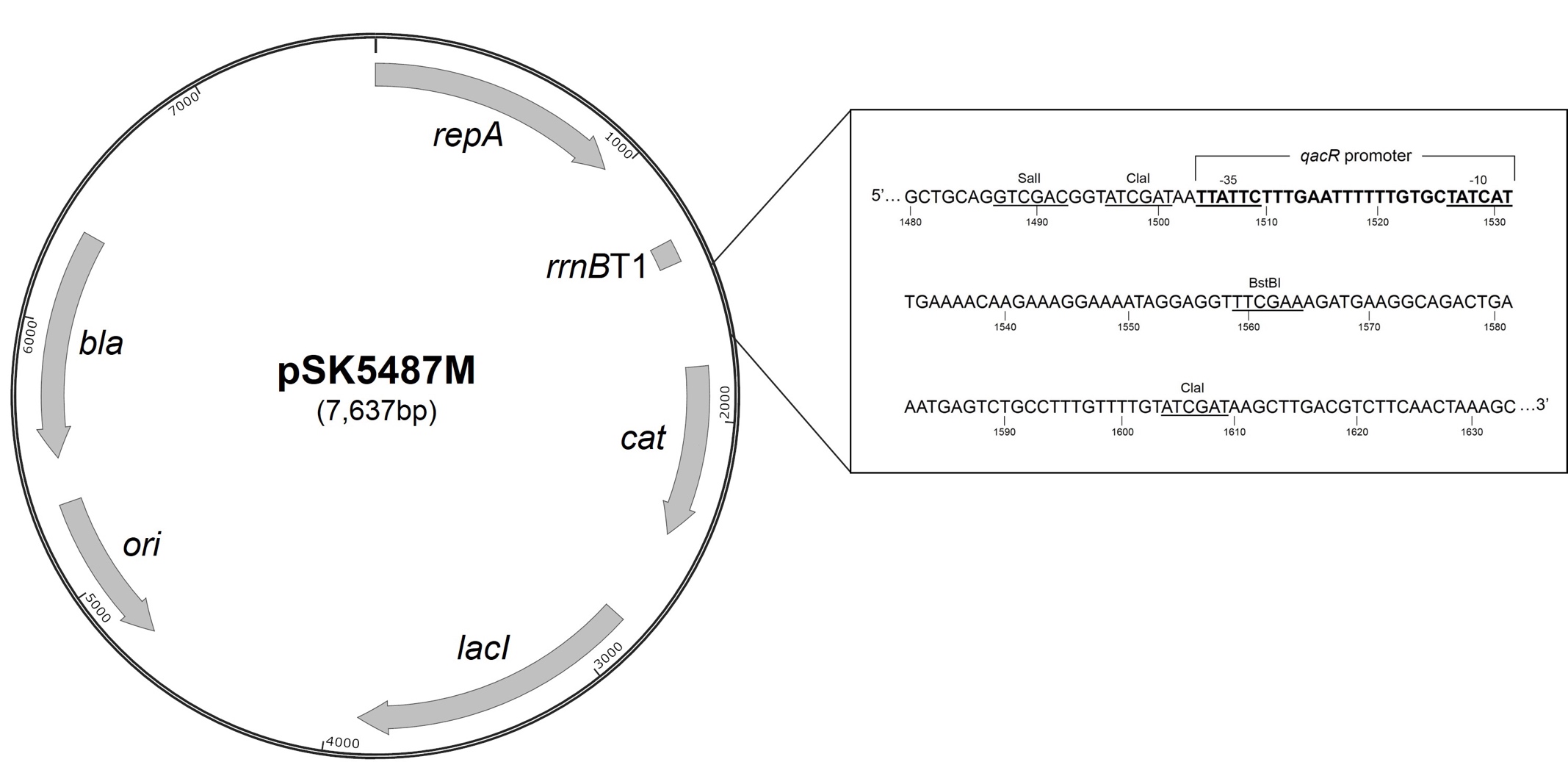
**

**Figure S1.** Plasmid pSK5487M.

Supplement: dkab063_Supplementary_Data [file dkab063_supplementary_data.docx]
